# Long non‐coding RNAs regulate treatment outcome in leukemia: What have we learnt recently?

**DOI:** 10.1002/cam4.6027

**Published:** 2023-05-06

**Authors:** Huiping Shi, Liang Gao, Weili Zhang, Min Jiang

**Affiliations:** ^1^ The First Affiliated Hospital of Soochow University Suzhou Jiangsu People's Republic of China; ^2^ Institutes of Biology and Medical Sciences Soochow University Suzhou Jiangsu People's Republic of China; ^3^ Department of Gastroenterology Xiangcheng People's Hospital Suzhou Jiangsu People's Republic of China; ^4^ Department of Oncology The First Affiliated Hospital of Soochow University Suzhou Jiangsu People's Republic of China

**Keywords:** hematopoietic stem cell transplantation, leukemia, long non‐coding RNA, targeted therapy, therapeutic resistance, treatment outcome

## Abstract

Leukemia is a group of highly heterogeneous and life‐threatening blood cancers that originate from abnormal hematopoietic stem cells. Multiple treatments are approved for leukemia, including chemotherapy, targeted therapy, hematopoietic stem cell transplantation, radiation therapy, and immunotherapy. Unfortunately, therapeutic resistance occurs in a substantial proportion of patients and greatly compromises the treatment efficacy of leukemia, resulting in relapse and mortality. The abnormal activity of receptor tyrosine kinases, cell membrane transporters, intracellular signal transducers, transcription factors, and anti‐apoptotic proteins have been shown to contribute to the emergence of therapeutic resistance. Despite these findings, the exact mechanisms of treatment resistance are still not fully understood, which limits the development of effective measures to overcome it. Long non‐coding RNAs (lncRNA) are a class of regulatory molecules that are gaining increasing attention, and lncRNA‐mediated regulation of therapeutic resistance against multiple drugs for leukemia is being revealed. These dysregulated lncRNAs not only serve as potential targets to reduce resistance but also might improve treatment response prediction and individualized treatment decision. Here, we summarize the recent findings on lncRNA‐mediated regulation of therapeutic resistance in leukemia and discuss future perspectives on how to make use of the dysregulated lncRNAs in leukemia to improve treatment outcome.

## 1 INTRODUCTION

Leukemia is a group of devastating malignant blood disorders characterized by high heterogeneity due to the differing origins of varying hematopoietic stem cells. Leukemia can be divided into acute myeloid leukemia (AML), chronic myeloid leukemia (CML), acute lymphoblastic leukemia (ALL), and chronic lymphocytic leukemia (CLL). Each type differs in incidence, pathogenesis, treatment, and prognosis. The worldwide number of new cases and deaths of leukemia in 2018 are 437,033 and 309,006, respectively.^1^ Current therapies for leukemia include chemotherapy, targeted therapy, hematopoietic stem cell transplantation, radiation therapy, and immunotherapy. As the most widely used treatment, chemotherapy eliminates leukemic cells for the induction, consolidation, and maintenance of remission. Targeted therapy such as tyrosine kinase inhibitors (TKIs) has greatly improved the outcome of those leukemia patients with aberrantly activated tyrosine kinases, a great example being the administration of imatinib in CML patients. Hematopoietic stem cell transplantation **(**HSCT) is a cornerstone in the treatment of leukemia as it replaces patients' leukemic cells with healthy donor hematopoietic cells, representing as a curative therapy. Despite these progresses in treatments, prognosis varies greatly in leukemia patients and is affected by multiple factors such as leukemia subtype, recurrent mutation, age, and therapeutic resistance. As a major challenge in the management of leukemia, treatment resistance greatly hinders the therapeutic efficacy of leukemia and leads to relapse or mortality. Some leukemia patients present primary unresponsiveness to therapeutics while others, despite initial response, eventually relapse, and progress. It is of urgent necessity to understand the pathogenesis and develop treatments to combat therapy resistance.

Long non‐coding RNAs (lncRNAs) are a group of non‐coding RNA molecules that are longer than 200 nucleotides. The lncRNAs show tissue‐ and cell‐type‐specific expression pattern and participate widely in many aspects of biological processes. LncRNAs are attracting growing attention as their important regulatory roles in physiological homeostasis and disease pathogenesis are being revealed. Indeed, lncRNAs have been shown to contribute to the pathogenesis, spreading, and therapeutic resistance of leukemia.^2^, ^3^ Some lncRNAs have been used as predictive biomarkers for drug resistance, while others may serve as targets to tackle the problem of therapeutic resistance. The study of lncRNAs sheds new light on the understanding and handling of therapy resistance.

The dysregulation of lncRNA expression in leukemia is related to multiple factors, such as genetic mutation, chromosomal rearrangement, and epigenetic regulation. For example, the lncRNA UCA1 has been shown to contribute to adriamycin resistance in AML^4^ and imatinib resistance in CML.^5^ UCA1 expression was upregulated by mutations in the *CEBPA* gene, which encodes CCAAT/enhancer‐binding protein‐α, in ~10% of the AML patients.^6^ Chromosome translocation t(15;17) in acute promyelocytic leukemia (APL) leads to the formation of PML‐RARα fusion protein, which has been shown to suppress the expression of the lncRNA NEAT1 in APL.^7^ Indeed, NEAT1 is related to resistance against alisertib and bortezomib in CML^8^ and multidrug resistance in ALL.^9^ In addition, epigenetic regulation affects the level of therapeutic resistance‐related lncRNAs in leukemia, such as MEG3, which is associated with cytarabine resistance in AML^10^ and imatinib resistance in CML.^11^ Of the 42 AML patients analyzed, MEG3 hypermethylation was identified in 20 AML patients (47.6%), which was related to significantly decreased overall survival in AML.^12^ Studies are ongoing to reveal the mechanisms of lncRNA dysregulation, which forms the basis of understanding lncRNA‐related drug resistance in leukemia and developing lncRNA‐based therapeutics.

**FIGURE 1:**
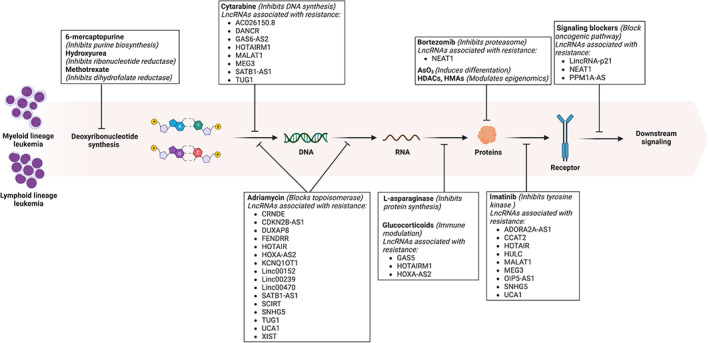
LncRNAs participate in therapeutic resistance development to some commonly used drugs in leukemia. Multiple treatments have been approved to treat myeloid and lymphoid leukemia, and these drugs target different biological processes. LncRNAs are found to participate in the development of therapeutic resistance to multiple drugs through various mechanisms. AsO_3_, arsenic trioxide; HDAC, histone deacetylase; HMA, hypomethylating agent; LncRNA, long non‐coding RNA. The image is created with BioRender.

Multiple mechanisms have been reported to confer therapy resistance in leukemia, including microRNA sponge,^13^ autophagy,^14^ apoptosis,^15^ DNA damage repair,^16^ oncogenic signaling,^17^ multi‐drug transporter/drug efflux,^18^ alterations of drug targets,^19^ metabolism of drugs,^20^ disruption of redox system,^21^ epigenetic regulation,^22^ chromosomal abnormalities,^23^ and increased stem cell populations.^24^ The emergence of these different resistance mechanisms is a result of interactions among various receptors, transporters, signal transducers, transcription factors, anti‐apoptotic proteins, etc. Among them, lncRNAs are a class of regulatory molecules that are found to confer therapeutic resistance in leukemia via multiple mechanisms, which are being vigorously studied (Figure 1). Recently, efforts have been made to functionalize lncRNAs in therapeutic resistance of leukemia by integrated genome‐wide CRISPRa approaches.^25^ By integrating analysis of computational, functional, and patient data, various resistance‐related lncRNAs involved in the regulation of cell‐cycle,^25^ survival/apoptosis,^25^ and cancer signaling pathways^25^ have been identified. Here, we summarize recent findings on lncRNA‐mediated regulation of treatment outcome in leukemia and discuss the future directions and challenges of the investigation on dysregulated lncRNAs in leukemia.

## 2 ROLES OF LncRNAs IN RESISTANCE TO LEUKEMIA TREATMENTS

### 2.1 Overview of lncRNAs in leukemia

Recent studies have revealed the multifaceted role of lncRNAs in the pathogenesis, spreading, and therapy response of leukemia.^26^, ^27^ LncRNAs exert their biological functions on leukemia via multiple mechanisms at both transcriptional and translational levels. Some lncRNAs regulate target gene expression in the nucleus via regulation of transcription factor binding or guiding chromatin‐modifying complexes. For example, HoxBlinc promotes leukemogenesis by controlling promoter chromatin accessibility via recruitment of the mixed lineage leukemia 1 complex and subsequent promoter H3K4me3 modification.^28^ In the cytoplasm, lncRNAs can function as competitive endogenous RNAs (ceRNAs) against certain microRNAs to regulate target mRNA translation. One such lncRNA is CRNDE, which promotes the progression of acute promyelocytic leukemia by sponging the miR‐181 family to regulate NOTCH2 expression.^29^ In addition, some lncRNAs are associated with recurrent causative mutations in leukemia, such as the increased expression of lncRNA MORRBID, which is correlated with recurrent mutations in two genes in AML: *TET2* ^30^ and *FLT3ITD*.^31^ Regarding treatment, while the managements of AML, CML, ALL, and CLL differ from each other, therapy resistance, either primary or secondary, is a common and unsolved problem among all subtypes. Recent studies enrich our understanding of how lncRNAs contribute to the development of therapeutic resistance.^2^, ^32^, ^33^ This review focuses on the novel findings of lncRNA‐mediated regulation of therapy resistance and treatment outcome in leukemia.

### 2.2 LncRNAs regulate therapeutic resistance in leukemia

#### 2.2.1 LncRNAs regulate therapeutic resistance in acute myeloid leukemia

Acute myeloid leukemia, the most common type of adulthood leukemia, has an incidence rate of 4.3 per 100,000 annually in the United States, with the occurrence rate increasing with age.^34^ The 5‐year survival rate is 35% ~ 40% for those under 60 years old and the rate decreases to 5% ~ 15% in those aged more than 60 years old.^35^ Chemotherapy remains the cornerstone for the induction, consolidation, and maintenance of remission in AML. However, the relapse rate is around 30% ~ 50% in young AML patients, which is even higher in elder AML patients.^25^, ^36^ The standard chemotherapy regimen in AML is based on the combination of nucleoside analogs (cytarabine and fludarabine) and anthracyclines (adriamycin, idarubicin, and daunorubicin). The well‐established “3 + 7 regimen” (3 days of daunorubicin and 7 days of cytarabine) is one such combination, which could lead to long‐term remission in 30% ~ 40% of the younger AML patients.^37^ After this initial induction, patients are given high dose cytarabine in the consolidation phase. The lncRNAs that contribute to chemotherapy resistance in AML and the corresponding mechanisms are summarized in Table 1.

**TABLE 1:** LncRNA contribution to chemotherapy resistance in acute myeloid leukemia and the corresponding mechanisms.

Chemotherapeutic	LncRNA	Target	Ref
Adriamycin	CRNDE	Wnt/β‐catenin pathway/MDR1	44
	HOTAIR	miR‐20a‐5p/WT1 axis DNMT3b upregulation and PTEN methylation	35, 48
	HOXA‐AS2	miR‐520c‐3p/S100A4 Axis	38
	KCNQ1OT1	miR‐193a‐3p/Tspan3	39
	Linc00152	Poly (ADP‐ribose) polymerase 1	45
	Linc00239	PI3K/AKT/mTOR	43
	SATB1‐AS1	miR‐580/ 2′‐5′‐oligoadenylate synthetase 2 axis	40
	SCIRT	miR‐21	41
	SNHG5	miR‐32/DNAJB9	42
	TUG1	miR‐34a AKT pathway	47, 55
	UCA1	miR‐125a/hexokinase 2 pathway	4
	XIST	miR‐29a/MYC	56
Cytarabine	AC026150.8	Splicing factor recruitment	57
	DANCR	miR‐874‐3P/ATG16L1 axis	50
	GAS6‐AS2	GAS6/TYRO3/AXL/MERTK pathway	25
	HOTAIRM1	Wnt/β‐catenin/PFKP pathway	49
	MALAT1	miR‐96	51
	MEG3	microRNA‐493‐5p/METTL3/MYC axis	10
	SATB1‐AS1	miR‐580/ 2′‐5′‐oligoadenylate synthetase 2 axis	40
	TUG1	miR‐655‐3p/CCND1 axis	58
Glucocorticoid	HOTAIRM1	ARHGAP18/RHOA/ROCK1 pathway	53

Adriamycin, also named doxorubicin, is a topoisomerase II inhibitor that serves as a commonly used chemotherapeutic drug in AML. Despite its efficiency in inducing remission, a substantial proportion of the AML patients develop resistance after the initial response, which leads to relapse and poor outcome. Multiple lncRNAs have been found to contribute to adriamycin resistance (Table 1). The reported lncRNA‐associated resistance mechanisms in leukemia can be divided into several categories: microRNA sponge,^4^, ^35^, ^38^, ^39^, ^40^, ^41^, ^42^ oncogenic signaling pathway activation,^43^ multi‐drug transporter‐mediated drug efflux,^44^ leukemia stem cell self‐renewal,^45^ and inhibition of tumor suppressors.^35^ MicroRNA sponging is the most common mechanism responsible for lncRNA‐mediated regulation of gene expression, in which lncRNAs compete with protein‐encoding mRNAs in the binding of shared target microRNAs. The lncRNA KCNQ1OT1 is found to adsorb miR‐193a‐3p and increase the expression of tetraspanin 3, which is required for the development and propagation of AML.^39^, ^46^ KCNQ1OT1 knockdown inhibited the proliferation, migration, and invasion of adriamycin‐resistant AML cells.^39^ The Wnt/β‐catenin and PI3K/AKT/mTOR pathways are among the classic oncogenic pathways, and both are reported to be altered by dysregulated lncRNAs and involved in chemoresistance of leukemia.^43^, ^44^, ^47^ Specifically, the lncRNA CRNDE was upregulated in AML patients treated with adriamycin‐based chemotherapy.^44^ CRNDE knockdown could sensitize AML cells to adriamycin by inhibiting the Wnt/β‐catenin pathway and reducing the expression of multidrug resistance protein (MDR1), which is an ATP binding cassette transporter that mediates the cellular efflux of multiple chemotherapeutic agents.^44^ The lncRNA linc00239 was one of the tumor promoters that are upregulated in AML patients.^43^ Linc00239 induced chemoresistance and prevented doxorubicin‐induced cellular apoptosis by activating the PI3K/AKT/mTOR pathway.^43^ TUG1 is another tumor‐promoting lncRNA that activated the AKT pathway in AML cells, and suppression of TUG1 could inactivate the AKT pathway to improve adriamycin cytotoxicity in AML cells.^47^ Regarding tumor suppressors, the lncRNA HOTAIR can epigenetically suppress the tumor suppressor PTEN (methylation) via DNMT3b upregulation, thus promoting drug resistance in AML.^48^

Cytarabine is an important nucleoside analog used for both induction and consolidation chemotherapy in AML. Like adriamycin, the Wnt/β‐catenin pathway is also critical in acquired resistance to cytarabine.^49^ HOTAIRM1 is one such lncRNA that regulates the Wnt/β‐catenin/PFKP signaling pathway to affect chemosensitivity to cytarabine.^49^ The lncRNA DANCR is shown to activate autophagy and confer cytarabine resistance in AML by the miR‐874‐3P/ATG16L1 axis.^50^ Similarly, through its microRNA sponge effect, knockdown of MALAT1 can enhance cytarabine chemosensitivity in AML by upregulating miR‐96.^51^ There are also some shared resistance mechanisms between adriamycin and cytarabine. The expression of lncRNA SATB1‐AS1 is increased in peripheral blood mononuclear cells derived from AML patients.^40^ By sponging miR‐580, SATB1‐AS1 upregulates OAS2 and contributes to the development of resistance against both adriamycin and cytarabine.^40^ Recently, large‐scale screening is made possible by CRISPR technology. One resistance‐associated lncRNA found in such way is GAS6‐AS2, which activates GAS6 and the downstream TYRO3/AXL/MERTK pathway to increase resistance to cytarabine.^25^

Glucocorticoids are used in combination with other therapeutics in AML to promote leukemic cell apoptosis.^52^ However, acquired resistance to glucocorticoids reduces the treatment efficacy in AML. HOTAIRM1 was found to be upregulated in glucocorticoid‐resistant AML cells and AML patients with poor prognosis.^53^ Indeed, glucocorticoid resistance in AML cells was caused by the anti‐apoptotic effect of HOTAIRM1 overexpression and its activation of the RHOA/ROCK1 signaling pathway.^53^

The clinical management of leukemia is limited by multiple factors, including patient tolerance to chemotherapy, emergence of therapeutic resistance and accumulation of pathological mutations, etc. This leads to studies that expand the available therapeutic choices so that treatments can be tailored for individuals. These novel drugs approved by the FDA include new‐generation kinase inhibitors, hypomethylating agents, anti‐apoptotic protein blockers, and monoclonal antibodies.^37^ However, the hyperplasticity of leukemia cells makes it hard to prevent the emergence of resistance to these newer treatments, and treatment failure has been reported.^54^ Studies are ongoing and whether lncRNAs are one of the culprits needs to be revealed.

#### 2.2.2 LncRNAs regulate therapeutic resistance in chronic myeloid leukemia

Chronic myeloid leukemia is developed due to the activation of a fused tyrosine kinase BCR‐ABL, which is caused by the translocation of the *ABL1* locus from chromosome 9 to the *BCR* locus on chromosome 22. Imatinib, a tyrosine kinase inhibitor targeting BCR‐ABL, is a standard therapy for newly diagnosed CML patients and has revolutionized the management of CML patients. According to the Glivec International Patients Assistance Program, the 7‐year survival was estimated to be as high as 87.7% in the imatinib‐treated 53,787 CML patients from 93 countries over 13 years.^59^ Unfortunately, resistance to imatinib develops in 15% ~ 20% of CML patients, which causes progression to the accelerated phase or blastic phase and increases the mortality rate.^60^, ^61^ The resistance mechanisms against imatinib that are related to lncRNAs are summarized in Table 2. Like resistance in AML, drug efflux mediated by MDR1 is an unsolved problem, which transports imatinib out of CML cells. The lncRNA UCA1 has been shown to increase expression of MDR1 by binding the shared miRNA response elements in miR‐16.^5^ Others found that MDR1 expression could be downregulated by HOTAIR knockdown in the imatinib‐resistant human CML cell lines, thus sensitizing leukemic cells to imatinib.^62^ In addition, HOTAIR might reduce CML cell apoptosis by activating the PI3K/AKT signaling pathway.^63^ Some other lncRNAs, such as ADORA2A‐AS1, MALAT1, MEG3, OIP5‐AS1, and SNHG5, also function as microRNA sponges to regulate the expression of genes related to therapy resistance.^11^, ^64^, ^65^, ^66^, ^67^

**TABLE 2:** LncRNA contribution to therapeutic resistance in chronic myeloid leukemia and the corresponding mechanisms.

Therapeutic	lncRNA	Target	Ref
Adriamycin	FENDRR	HuR/FENDRR/miR‐184	71
	Linc00470	METTL3/PTEN/autophagy	70
Alisertib and bortezomib	NEAT1	ATP‐binding cassette G2	8
Imatinib	ADORA2A‐AS1	miR‐665/TGFBR1 and ABCC2	64
	CCAT2	NA	73
	HOTAIR	MDR1, PI3K/AKT pathway	62, 63
	HULC	miR‐200a, PI3K/AKT pathway	74
	MALAT1	miR‐328	65
	MEG3	miR‐21	11
	OIP5‐AS1	miR‐30e‐5p/ATG12/autophagy	66
	SNHG5	miR‐205‐5p	67
	UCA1	miR‐16/MDR1	5

To overcome the resistance against imatinib, new generations of TKIs, such as bosutinib, dasatinib, nilotinib, and ponatinib, are developed. Unfortunately, drug resistance against these novel agents has also been reported.^68^ Alisertib is an inhibitor for aurora A kinase, which is shown to be upregulated by BCR‐ABL in CML via AKT signaling.^69^ Alisertib effectively inhibited the proliferation of CML cells harboring the imatinib‐resistant BCR‐ABL T315I mutation both in vitro and in vivo.^69^ Resistance to alisertib has been found to be associated with the expression of the drug resistance‐conferring ATP‐binding cassette G2, and the lncRNA NEAT1 could sensitize CML cells to alisertib by inhibiting the expression of ATP‐binding cassette G2 in leukemia.^8^ Overall, future studies are warranted to reveal potential lncRNAs involved in resistance to these newer TKIs.

Hyper‐CVAD (cyclophosphamide, vincristine sulfate, adriamycin, dexamethasone) in combination with TKI is one of the regimens used in those with CML that progressed into blastic phase. Resistance against one of the components, adriamycin, has been reported. Linc00470 regulates the RNA methyltransferase METTL3 to decrease the stability of PTEN and activate AKT, thus conferring chemoresistance in CML.^70^ In addition, adriamycin resistance can be attenuated via MDR1 suppression by lncRNA FENDRR‐mediated sponging of HuR and miR‐184 in CML cells.^71^ Other novel therapies are also being proposed for the treatment of CML. For example, bortezomib is a proteasome inhibitor and has been found to be cytotoxic against not only TKI‐insensitive stem and progenitor CML cells but also CML cells with TKI resistance‐conferring BCR‐ABL mutations.^72^ ATP‐binding cassette G2 has been shown to reduce the efficacy of bortezomib, which could be inhibited by lncRNA NEAT1.^8^

#### 2.2.3 LncRNAs regulate therapeutic resistance in acute lymphoblastic leukemia

ALL is developed from the lymphoid lineage and more likely to occur in children and young adults. Chemotherapy is the mainstay therapy for ALL. For those with the fused tyrosine kinase BCR‐ABL, TKI is also typically included in the treatment. Among the chemotherapeutic drugs, lncRNA‐related resistance has been reported for adriamycin and glucocorticoids (Table 3). The PI3K/AKT/mTOR signaling pathway is important for the pathogenesis and chemoresistance of B‐cell ALL.^75^ PIK3CA is regulated by the lncRNA DUXAP8 via its competitive binding of miR‐29a, and DUXAP8 knockdown can reduce the PI3K/AKT/mTOR signaling and restore chemosensitivity to adriamycin.^76^ Regarding glucocorticoids, overexpression of GAS5, a potent glucocorticoid receptor riborepressor, leads to poor treatment outcome in pediatric B‐cell ALL.^77^ In addition, HOXA‐AS2 is found to confer glucocorticoid resistance by upregulating HOXA3 and activating the EGFR/Ras/Raf/MEK/ERK signaling pathway, thus increasing cell proliferation, and reducing cell apoptosis.^78^

**TABLE 3:** LncRNA contribution to therapeutic resistance in acute lymphoblastic leukemia and the corresponding mechanisms.

Therapeutic	LncRNA	Target	Ref
Adriamycin	CDKN2B‐AS1	miR‐335‐3p/TRAF5 axis	81
	DUXAP8	miR‐29a/ PIK3CA	76
Glucocorticoid	GAS5	Glucocorticoid/glucocorticoid receptor axis	77
	HOXA‐AS2	EGFR/Ras/Raf/MEK/ERK pathway	78
Multidrug	NEAT1, MALAT1	miR‐335‐3p/ABCA3	9

About 10%–15% of the childhood and 25% of the adulthood ALL cases are derived from T‐cell progenitors, and their prognosis is poorer than those with B‐cell ALL, partly due to higher rates of chemoresistance and relapse.^79^ CDKN2B‐AS1 promotes the resistance against adriamycin via the miR‐335‐3p/TRAF5 axis in pediatric T‐cell ALL.^81^ The lncRNA PPM1A‐AS can regulate important signaling molecules including Notch4, STAT3, and AKT, to promote cell proliferation and inhibit cell apoptosis.^80^

#### 2.2.4 Therapeutic resistance in chronic lymphocytic leukemia and role of lncRNAs

CLL is one type of mature B‐cell neoplasms and it has a non‐leukemic manifestation named as small lymphocytic lymphoma.^82^ CLL is an indolent malignancy that is more commonly diagnosed in elder adults.^83^ CLL treatments include chemotherapy, Bruton's tyrosine kinase inhibitors, PI3K inhibitors, monoclonal antibodies, anti‐apoptotic protein inhibitors, and immune checkpoint inhibitors.^84^ The therapeutic resistance in CLL is largely unknown and being intensively studied. While there is evidence showing the contribution of multiple microRNAs in treatment refractoriness,^85^ it remains understudied whether and how lncRNAs regulate chemosensitivity in CLL. In fact, several agents used in CLL are also effective therapeutics for other cancer types, in which resistance mechanisms related to lncRNAs have been reported.^86^, ^87^, ^88^ One such example is ibrutinib, an irreversible inhibitor of Bruton tyrosine kinase. Ibrutinib was initially approved by FDA in patients with mantle cell lymphoma, and later, it was approved as a first‐line treatment for patients with CLL.^86^ Therapeutic resistance remains a challenge in CLL treatment, especially in those treated with single‐agent ibrutinib: the rates of CLL progression are 15.5% and 20.9% for treatment‐naïve and relapsed/refractory CLL patients, respectively.^86^, ^87^, ^88^ The lncRNA LINK‐A has been shown to increase the anti‐apoptotic BCL2 by activating AKT signaling to confer ibrutinib resistance in mantle cell lymphoma.^89^ However, whether lncRNA LINK‐A or other lncRNAs contribute to the resistance against ibrutinib or other TKIs in CLL remains to be studied.

## 3 LncRNAs REGULATE HEMATOPOIETIC STEM CELL TRANSPLANTATION OUTCOME IN LEUKEMIA

As a curative therapy, hematopoietic stem cell transplantation **(**HSCT) remains a cornerstone in the management of leukemia. One of the major challenges for successful HSCT is acute and chronic graft versus host disease (GvHD). The importance of B/T lymphocytes and dendritic cells in the pathogenesis of GvHD have been demonstrated.^90^, ^91^, ^92^ Recent evidence indicates that lncRNAs affect GvHD occurrence by regulating these cells. Lnc‐MAF4 and IFNG‐AS1 are related to IFNγ production and TH1 cell differentiation, and they were found to be higher in acute GvHD patients at day 28 and 52 ± 8 when compared to non‐GvHD patients.^93^ The same group also found higher lnc‐DC levels at day 28 after HSCT in patients with a GvHD than non‐GvHD patients.^94^ There are also studies exploring the role of lncRNAs in chronic GvHD by microarray analysis.^95^ For example, the lncRNAs NONHSAT142151, NONHSAT040475, and FR118417 have been shown to correlate with B cell receptor signaling^95^ and chronic GvHD.^95^ HSCT efficiency is also determined by the homing, proliferation, and differentiation of transplanted stem or progenitor cells.^96^ Competitive transplantation experiments revealed that the lncRNA Spehd is required for multilineage differentiation, and its silencing led to deficient oxidative phosphorylation pathway in common myeloid progenitors.^97^ Further studies are needed to reveal how lncRNAs regulate HSCT outcome in leukemia and identify potential targets to promote successful engraftment and long‐term survival of the transplant.

## 4 FUTURE PERSPECTIVES ON lncRNA‐BASED STRATEGIES TO IMPROVE TREATMENT OUTCOME PREDICTION IN LEUKEMIA

Although multiple prognostic factors including leukemia type, age, white blood cell count, cytogenetics, and recurrent mutations have been identified, the outcome prediction and risk stratification in leukemia patients are still not optimal and need improvement. Given lncRNAs' important role in regulating leukemia patient outcome, there are studies to investigate the potential of incorporating lncRNAs to improve the current prediction model for drug resistance and exploiting them as potential targets to overcome therapeutic resistance.^98^, ^99^ Indeed, the lncRNA landscape has been characterized in multiple leukemia types and corresponding lncRNA scores have been developed for outcome prediction. The lncRNA score was able to stratify older cytogenetically normal AML patients into longer and shorter disease‐free survival and overall survival groups.^26^ Similarly, juvenile myelomonocytic leukemia patients with favorable and unfavorable lncRNA scores had significantly different overall survival and event‐free survival after HSCT.^100^ Wang et al. found a higher expression level of the lncRNA KIAA0125 in the bone marrow of 347 de novo AML patients. When using KIAA0125 expression to stratify standard chemotherapy‐treated AML patients, those with higher KIAA0125 expression had lower complete remission rate, shorter overall survival, and disease‐free survival than the low‐expression group.^101^ The HOTAIR is one of the well‐characterized oncogenic lncRNAs, whose expression was found to be significantly higher in AML patients than normal controls. Interestingly, the post‐treatment HOTAIR expression level was found to be correlated to relapse‐free survival and might predict relapse susceptibility.^102^ The lncRNA CCAT2 was upregulated in the peripheral blood of CML patients and associated with therapy response, as the enhanced expression of CCAT2 at diagnosis indicates imatinib resistance in CML.^73^ A higher level of lncRNA TUG1 was found to be negatively correlated with complete remission within 4 weeks, total complete remission, and allogeneic HSCT achievement in adult Ph^−^ ALL.^103^ Therefore, correlations between leukemia patient treatment outcome and lncRNA expression/function have been identified by multiple studies. In the future, it would be helpful if specificized algorithms integrating lncRNAs and other prognostic factors are developed for each leukemia subtype to predict patient outcome and treatment response.

## 5 TARGETING lncRNAs TO REDUCE DRUG RESISTANCE IN LEUKEMIA

Due to the important role of ncRNAs in tumorigenesis and therapeutic resistance development, efforts have been made to develop ncRNA‐targeting therapies for cancers. Among these ncRNAs, miRNAs are better studied, and some therapeutics based on miRNAs, including miRNA mimics and anti‐miRNA oligonucleotides, are being tested in patients with diabetes, liver diseases, or keloid in clinical trials.^104^ Though miRNA mimics and anti‐miRNA oligonucleotides have not been tested clinically in tumors, numerous pre‐clinical studies are ongoing to determine if they can effectively overcome drug resistance in multiple cancers including leukemia.^105^, ^106^, ^107^

By contrast, there is no lncRNA‐based therapy that is approved or under clinical testing as the studies of lncRNAs are emerging only in recent years, which is much later than miRNAs.^104^ Nevertheless, lncRNAs have huge potential to be used as therapeutic targets in cancer due to their specific expression pattern and wide‐ranging functions. There are multiple strategies to modulate lncRNAs in cancer, including (a) transcriptional inhibition or upregulation using CRISPR/Cas9 technology; (b) post‐transcriptional lncRNA degradation mediated by antisense oligonucleotides or small interfering RNAs; (c) steric inhibition of lncRNA‐protein interactions (d) disruption of lncRNA secondary structure.^108^ The lncRNA MALAT1 plays a crucial role in leukemia therapeutic resistance, as it has been found to contribute to cytarabine resistance in AML,^51^ imatinib resistance in CML,^65^ and multidrug resistance in ALL.^9^ The triple‐helix structure of MALAT1 is shown to be targetable by small molecules.^109^, ^110^ These small molecules have been tested in the mammary tumor organoid model but not in leukemia.^110^ Given the important role of MALAT1 in leukemia, it would be interesting to test these small molecules in the future and determine whether they are effective in reducing drug resistance in leukemia.

## 6 CONCLUSIONS

The role of lncRNAs in the regulation of treatment response in leukemia is undoubtedly important. Treatment options for leukemia are expanding as new kinase inhibitors, hypomethylating agents, anti‐apoptotic protein blockers, and monoclonal antibodies get approved to be used in leukemia patients. Resistance to some of these agents has been noticed, which is related to drug transport, metabolism, DNA repair, and apoptosis regulation. As summarized above, great progress has been made to understand lncRNA‐mediated therapy resistance in leukemia, but much is left to be studied as how and why patients become unresponsive to novel anti‐leukemic drugs are not fully clear. As resistance‐promoting and inhibiting lncRNAs are being identified, it would be possible to exploit them as potential targets and regulate their expression to combat therapy resistance in leukemia. Furthermore, lncRNAs can be used as biomarkers to optimize risk stratification and predict treatment response. Additionally, lncRNAs can aid in individualized treatment planning for leukemia patients as they can be used to identify patients who will likely to benefit from certain therapies. These are areas that are of great importance but understudied, thus warranting further investigation.

## AUTHOR CONTRIBUTIONS

**Huiping Shi:** Conceptualization (equal); data curation (equal); formal analysis (equal); visualization (equal); writing – original draft (equal). **Liang Gao:** Conceptualization (equal); data curation (equal); formal analysis (equal); visualization (equal). **Weili Zhang:** Conceptualization (equal); visualization (equal); writing – original draft (equal). **Min Jiang:** Conceptualization (equal); funding acquisition (lead); supervision (lead); writing – review and editing (equal).

## Data Availability

Data sharing is not applicable to this article as no new data were created or analyzed in this study.
